# FGF-mediated establishment of left-right asymmetry requires Rab7 function in the dorsal mesoderm in *Xenopus*

**DOI:** 10.3389/fcell.2022.981762

**Published:** 2022-08-29

**Authors:** Jennifer Kreis, Celine Marie Camuto, Carolin Charlotte Elsner, Sebastian Vogel, Philipp Vick

**Affiliations:** Department of Zoology, University of Hohenheim, Stuttgart, Germany

**Keywords:** Rab7, Fgf, left-right asymmetry, left-right organizer, mesoderm, *Xenopus*

## Abstract

Gastrulation denotes a very important developmental process, which includes significant structural tissue rearrangements and patterning events that shape the emerging vertebrate organism. At the end of gastrulation, the three body axes are spatially defined while the left-right axis still lacks any molecular or morphological polarity. In most vertebrates, this is established during neurulation by a symmetry breaking LR organizer. However, this mesoderm-derived structure depends on proper induction and specification of the mesoderm, which in turn requires involvement of several signaling pathways. Endocytosis and the endosomal machinery offer manifold platforms for intracellular pathway regulation, especially late endosomes claim increasing attention. The late endosomal regulator Rab7 has been linked to mesoderm specification during gastrulation. Distinct axial defects due to compromised dorsal mesoderm development in *rab7*-deficient *Xenopus* embryos suggested a requirement of Rab7 for FGF-dependent mesoderm patterning and LR asymmetry. Here we specifically addressed such a role of Rab7, demonstrating a functional requirement for LR organizer development and symmetry breakage. Using different FGF/MAPK pathway components we show that Rab7 participates in dorsal mesoderm patterning. We suggest a hierarchical classification of Rab7 upstream of MAPK-dependent mesoderm specification, most probably at the level of the small GTPase Ras. Thus, this study affords an insight on how the Rab7-regulated endosomal machinery could participate in signal transduction to enable correct mesoderm specification and left-right asymmetry.

## Introduction

All vertebrates share asymmetric arrangement of their visceral organs with respect to the left-right (LR) body axis. In contrast to early determination and establishment of anteroposterior (AP) and dorsoventral (DV) axes before and during gastrulation, molecular and morphological asymmetries between left and right are established during later neurula stages in most vertebrates. Except for a few taxa, these asymmetries are induced by a LR organizer (LRO), which is represented by a monociliated epithelium inside the developing archenteron. Functionally, the LRO generates an extracellular fluid-flow from the right to the left side of the embryo. This flow triggers a complicated and only partially understood molecular cascade in the lateral/somitic LRO (sLRO), a sensory area flanking the central, flow-generating part (cLRO). This activates the Nodal signaling cascade exclusively in the left lateral plate mesoderm, finally coordinating morphological asymmetries (Grimes and Burdine, 2017; Blum and Ott, 2018a; Hamada, 2020). One distinct feature of a LRO is the temporally restricted existence and its mesodermal fate, embedded in the future endodermal gut epithelium. Probably best analyzed in the frog *Xenopus*, by the end of neurulation, cLRO will integrate in the notochord and hypochord, while the sLRO is destined to contribute to the somites. Reflecting its mesodermal fates, the origin of the LRO can be traced back to the superficial mesoderm (SM) during gastrulation, which represents the outer epithelial lining of the posterior (i.e., trunk) organizer (Blum and Ott, 2018b; Blum et al., 2014; Cooper and D'Amico, 1996; Shook et al., 2004). During early gastrulation, the forkhead transcription factor *foxj1*, a master control gene for motile cilia, highlights this crescent-shaped area (Stubbs et al., 2008; Blum and Ott, 2018b). But in contrast to later neurula stages, “classical” mesodermal marker genes seem to be inactive in the superficial area at that timepoint. However, disrupting FGF signaling during gastrulation inhibited *foxj1* expression or later sLRO formation, depending on the type and timepoint of treatment (Sempou et al., 2018; Schneider et al., 2019). The FGF pathway is known to be required for (deep) axial and paraxial mesoderm development, and blocking FGF receptor activation from early gastrulation onwards specifically inhibits MAPK-dependent dorsal expression of mesodermal genes, resulting in massive dorsal/axial phenotypes (Amaya et al., 1993; Christen and Slack, 1999; Fletcher and Harland, 2008; Dorey and Amaya, 2010; Kjolby et al., 2019).

Regulation of signaling pathways can occur in diverse ways, enabling multi-level inputs and precise fine-tuning. One way of regulating intracellular signal transduction is offered by endocytic membrane trafficking. Translocation of activated receptors through the endocytic system can lead to their final degradation in lysosomes. However, individual endosomal compartments are now considered as signal generating or maintaining platforms, in some cases also a lysosome-related late endosome (LE; Platta and Stenmark, 2011; Sigismund et al., 2012). Trafficking within the endosomal system is regulated by Rab proteins. Transient attachment of these small GTPases to their specific endosomal compartment orchestrates effector recruitment, finally regulating functionality (Stenmark, 2009). In our recent work we analyzed the role of the late endosome master regulator Rab7 for early embryonic development in *Xenopus*. We demonstrated that *rab7* was required for involution and axial elongation during gastrulation by regulating mesodermal specification (Kreis et al., 2021). While organizer induction and DV patterning were unaltered in Rab7-deficient embryos, *foxj1* expression in the SM was strongly inhibited. Specification of the somitic lineage was blocked as well, as demonstrated by loss of marker genes in the paraxial mesoderm (*myod1*, *myf5, tbx6*). As these tissues represent precursors required for LRO morphogenesis after gastrulation, our observations pointed towards an involvement of Rab7 in LR development, potentially in an FGF-dependent manner.

Here we analyzed a putative role of Rab7 for LR development in context of FGF-dependent mesoderm patterning. Defined knockdown in the paraxial mesodermal lineage prevented LRO sensor specification and resulted in laterality effects, independent of general mesoderm induction or gastrulation. The lack of LR sensor specification was most likely caused by a loss of the paraxial identity during gastrulation, which could be restored by exogenous activation of MAPK signaling or the downstream Ets2 transcription factor. The same rescue effect could be observed when general mesoderm induction was blocked by Rab7 knockdown. Using animal caps we show that Rab7 was required for both, FGF-dependent elongation and FGF-induced mesodermal identity, suggesting a general role of Rab7 in FGF/MAPK-dependent dorsal mesoderm specification in *Xenopus*.

## Results

### Rab7 is necessary for correct LR axis development

**FIGURE 1:**
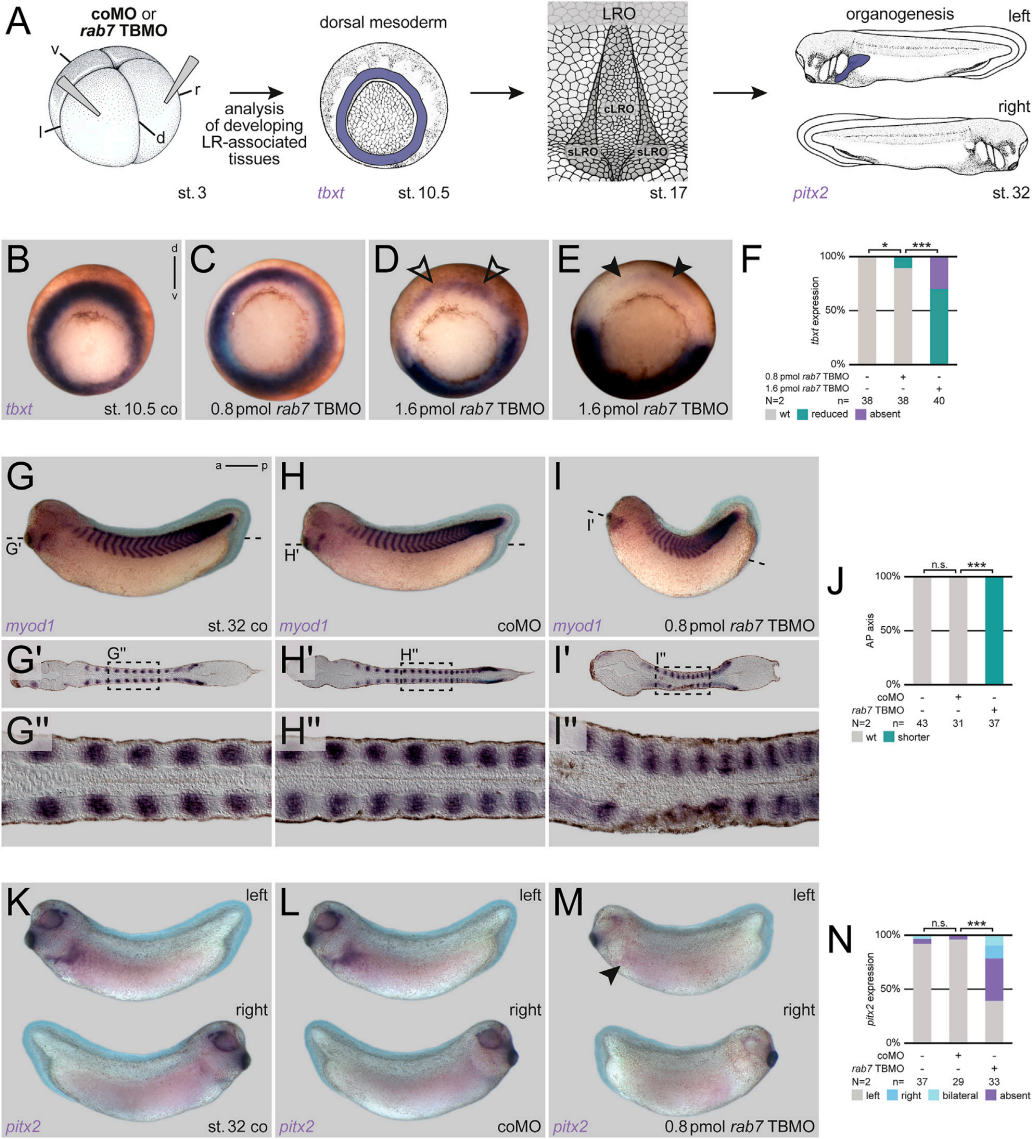
Rab7 is necessary for correct LR axis development. **(A)** Experimental setup for the shown experiments: Injections at four-cell stage (left) targeted the dorsal mesoderm to analyze mesoderm specification (*tbxt*; middle-left) at gastrula stages, a prerequisite for the formation of the dorsal mesoderm derived LRO (middle-right) in wild type situation during neurula stages, which in turn induces LR asymmetric features (here *pitx2* expression; right) at tailbud stages. **(B)** Wild type circular *tbxt* expression of st. 10.5 control specimen, **(C)** injection of reduced amounts of *rab7* TBMO did not alter *tbxt* expression, **(D)** whereas higher doses reduced transcripts (black outlined arrowheads) or **(E)** abolished them in the targeted dorsal lineage (black arrowheads). **(F)** Quantification of *tbxt* expression. **(G)** Uninjected st. 32 tadpole, or specimen injected with **(H)** coMO depicted wild type axis elongation compared to **(I)** shortened axis of *rab7* TBMO treated specimens. **(G′–I′)** Please note in frontal sections through somitic lineage that all specimen showed *myod1* expression in somites along AP-axis. **(G′′–I′′)** Magnification of *myod1* expressing somites as indicated in dashed boxes, respectively. Please note altered somite shapes in morphants. **(J)** Quantification of axis phenotype. **(K)** Left-sided *pitx2* expression in untreated st. 32 tadpoles and **(L)** coMO injected ones. **(M)** Lost left-sided *pitx2* expression upon *rab7* knockdown (black arrowhead). **(N)** Quantification of *pitx2* expression. a, anterior; cLRO, central LRO; co, control; coMO, control Morpholino oligomere; d, dorsal; l, left; LRO, left-right organizer; N, number of experiments; n, number of evaluated embryos; n.s., not significant; p, posterior; pmol, picomole; r, right; sLRO, somitic LRO; st., stage; TBMO, translation blocking Morpholino oligomere; v, ventral; wt, wild type.

Our previous analyses of the role of Rab7 in mesoderm patterning during gastrulation suggested a regulatory role for pre-patterning and specification of the LRO or its precursor tissues as well (Kreis et al., 2021). To address the hypothesized impact of Rab7 deficiency on later LRO function and LR asymmetry, we injected *Xenopus* embryos at the four-cell stage with reduced amounts of a *rab7* translation blocking Morpholino oligomere (TBMO; cf. Kreis et al., 2021) into the dorsal lineage, i.e. targeting the future LRO (see Figure 1A for experimental setup; cf. Blum et al., 2009). In this setting, the majority of morphant embryos showed wild type *tbxt* expression (Figures 1B,C,F), indicating normal mesoderm induction, which was not the case for high-dose injections (Figures 1D–F). Gastrulation and blastopore closure proceeded apparently normal and most specimens could be raised to tailbud stages. By late tailbud stages, these embryos developed mild phenotypes with reduced anterior-posterior elongation, which became evident when analyzed by ISH for *myod1* to highlight somites and presomitic mesoderm (Figures 1G–J). In frontal sections somites were distinguishable but appeared compressed, supporting specific requirement of Rab7 for normal development in the somitic lineage (Figures 1G'–I''). Importantly, when such embryos were stained for LR marker gene *pitx2*, the majority exhibited laterality defects (Figures 1K–N). From these experiments we concluded that Rab7 is required for paraxial mesoderm specification and for LR axis formation before symmetry breakage.

### Lack of LR organizer sensor specification upon Rab7 knockdown

**FIGURE 2:**
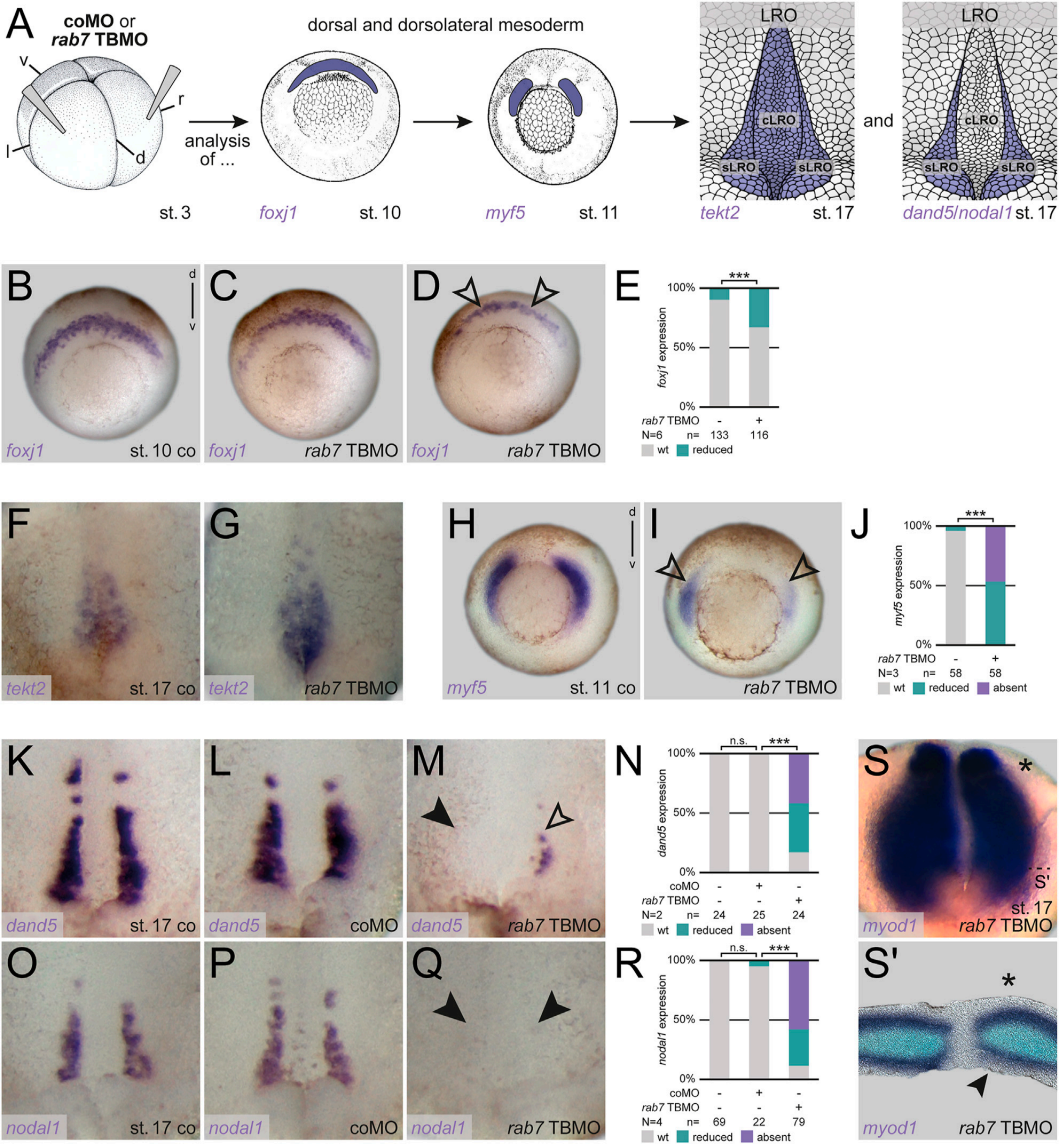
Rab7 knockdown resulted in loss LRO sensor tissue. **(A)** Experimental setup for the shown experiments: Injections at four-cell stage (left) targeted the dorsal/dorsolateral mesoderm to analyze superficial mesoderm induction (*foxj1*; middle) or somitic mesoderm specification (*myf5*; middle) at gastrula stages, following analyses of general LRO formation (*tekt2*; right) or sLRO specification (*dand5*/*nodal1*; right) during neurulation. **(B)** Wild type crescentic *foxj1* transcripts were **(C)** unaltered or **(D)** mildly affected by low dose *rab7* TBMO injections (black outlined arrowheads). **(E)** Quantification of *foxj1* expression. **(F)** Expression domain of *tekt2* in the LRO of st. 17 embryos, **(G)** depicted slightly stronger posterior and reduced anterior signals upon low dose *rab7* TBMO treatment. **(H)** Wild type dorsolateral *myf5* expression **(I)** was significantly reduced in *rab7*-morphant embryos (black outlined arrowheads). **(J)** Quantification of *myf5* expression. **(K,O)** Expression of *dand5* and *nodal1* in the somitic domains of the LRO of control and **(L,P)** coMO injected specimen, respectively. **(M)** Transcripts of *dand5* or **(Q)** *nodal1* were lost (black arrowheads) or severely reduced (black outlined arrowheads) in *rab7*-deficient embryos (low dose TBMO). **(N,R)** Quantification of *dand5* and *nodal1* expression, respectively. **(S)** Left-sided unilateral knockdown (marked by asterisk) of low dose *rab7* TBMO in *myod1* expressing LRO, **(S′)** section revealed lost superficial expression of *myod1* at injection side (black arrowhead). cLRO, central LRO; co, control; coMO, control Morpholino oligomere; d, dorsal; l, left; LRO, left-right organizer; N, number of experiments; n, number of evaluated embryos; n.s., not significant; r, right; sLRO, somitic LRO; st., stage; TBMO, translation blocking Morpholino oligomere; v, ventral; wt, wild type.

First, to understand the basis of the LR asymmetry phenotype, we analyzed *foxj1* at early gastrula stages to check SM specification, and *tekt2*, a Foxj1 target gene highlighting the differentiated LRO during neurulation (see Figure 2A for experimental setup; Stubbs et al., 2008). We have previously shown that inhibition of Rab7 (using higher MO concentrations) strongly blocked *foxj1* expression, i.e., LRO precursor formation (Kreis et al., 2021). In our low-dose MO approach used here, morphant embryos always showed activation of *foxj1* during gastrulation, but approximately one third of them displayed reduced expression (Figures 2B–E). In these cases, this reduction was detectable as an animal-to-vegetal thinner domain (Figure 2D). Similarly, *tekt2* showed altered expression patterns in some morphant embryos, with stronger posterior and reduced anterior signals, but activation itself was not inhibited (Figures 2F,G). Thus, in this experimental setup (i.e., with partial depletion of Rab7), a mild effect was observed on general LRO specification.

Asymmetric Nodal cascade induction is initiated by the somitic part of the LRO (Figure 2A). Therefore, we analyzed the somitogenic factor *myf5* during gastrulation, which showed strong reductions under these conditions, indicating a high sensitivity of the paraxial tissues to a loss of Rab7 (Figures 2H–J). This strong inhibition of *myf5*, which has been implicated in LR symmetry breakage very recently (Tingler et al., 2022), suggested a potential impact on LRO sensor specification as well. Thus we wondered whether sLRO cells were specified correctly, i.e., expressed characteristic marker genes (Figure 2A). Reflecting the high percentage of altered *pitx2* expression, the majority of morphant embryos showed reduction or loss of both, *dand5* and *nodal1* in the sLRO (Figures 2K–R). As the lateral cells of the LRO are of somitic fate, we analyzed unilateral morphants for *myod1*, a somitic marker known to be selectively expressed in the lateral LRO (Shook et al., 2004; Schweickert et al., 2010). Similar to internal control halves, *rab7* TBMO injected LRO halves showed expression of *myod1* in the presomitic mesoderm (Figure 2S). However, the epithelial expression in the sLRO was lost or reduced on the morphant side of most embryos (Figure 2S′). These results support the conclusion that dorso-lateral loss of Rab7 caused a failure of sLRO specification, preventing correct Nodal cascade induction.

### Loss of Rab7 blocks dorsal mesoderm specification upstream of MAPK activation

As for the axial and paraxial deep mesoderm, induction of the SM and specification of the lateral sLRO have been shown to depend on FGF receptor-derived patterning signals during blastula/gastrula stages (Sempou et al., 2018; Schneider et al., 2019). Therefore, AP/somite (*myf5*/*myod1*; Figures 1, 2) and laterality (*pitx2c*/*foxj1*/*nodal1/dand5*; Figures 1, 2) phenotypes presented above both possibly reflect a partial loss of FGF signaling. In line with our stronger effects on the sLRO, the paraxial mesoderm has been reported to be more sensitive to FGF inhibition (Amaya et al., 1993). Indeed, in the same injection setup (reduced doses, somitic lineage targeted), loss of Fgfr1 phenocopied the loss of Rab7 in tailbud stages (Supplementary Figures S1A–C; cf. also Schneider et al., 2019). Further, our recent findings of defects in mesoderm patterning and subsequent gastrulation movements after loss of Rab7, which resulted in severe dorsal/posterior truncations at tailbud stage, were also highly reminiscent of a loss of FGF receptor 1 mediated signaling (Amaya et al., 1991, 1993; Kreis et al., 2021). Based on these parallels, we hypothesized that Rab7 was required for FGF-mediated specification and/or maintenance of mesodermal and somitic fates during gastrulation, i.e., upstream of MAPK cascade activation. Such a function would sufficiently explain the mesodermal and laterality-related phenotypes after dorsal-specific loss of Rab7. To address this hypothesis, we analyzed the role of Rab7 in context of MAPK signaling during gastrulation.

**FIGURE 3:**
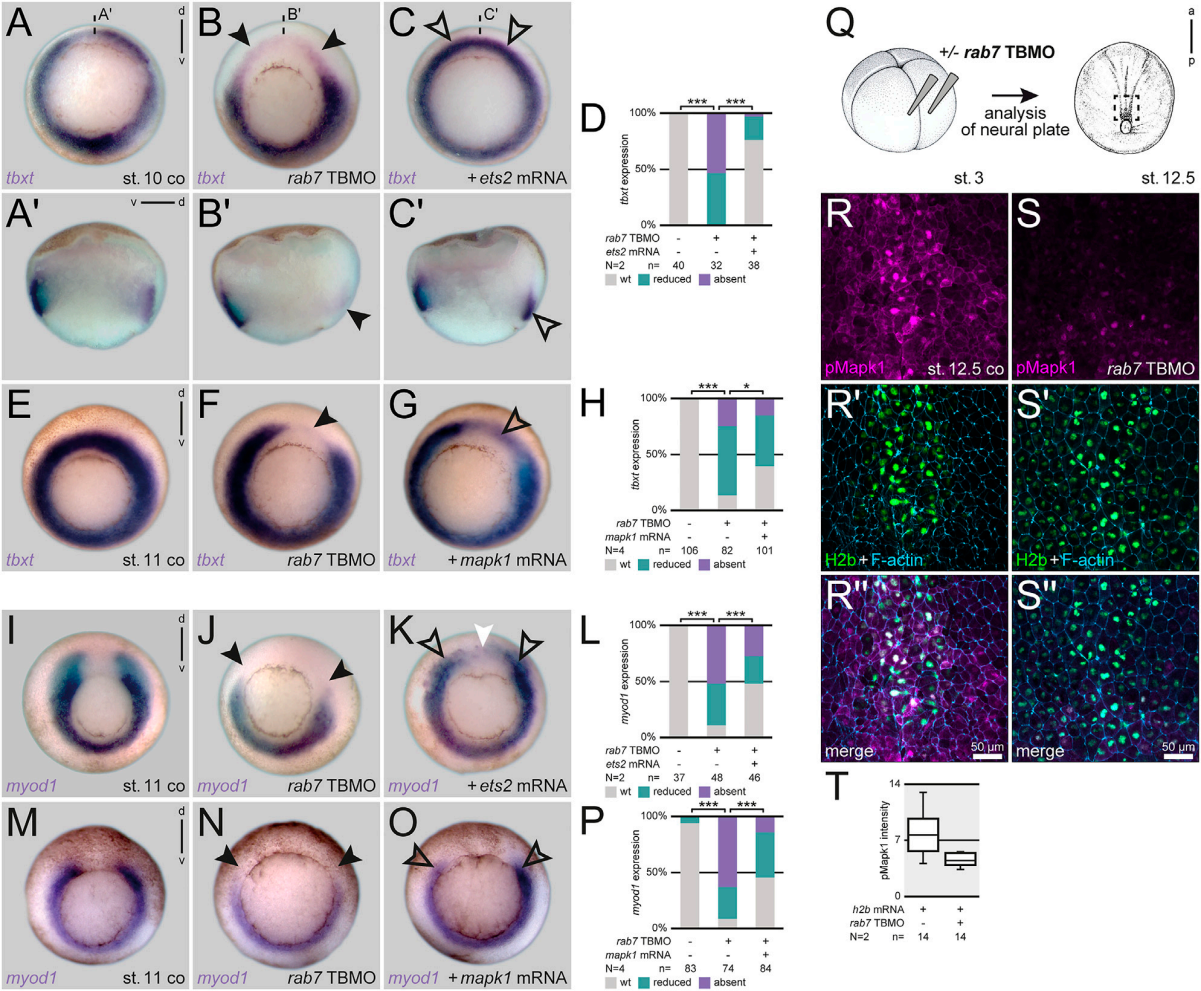
Loss of Rab7 blocks dorsal mesoderm specification upstream of Mapk activation. **(A)** Uniform circular appearance of *tbxt* expression of st. 10 *Xenopus* embryos **(B)** faded due to dorsal knockdown of *rab7* (black arrowheads). **(C)** Rescue injection of *ets2* mRNA restored expression almost back to wild type level (outlined black arrowheads). **(A′–C′)** Panels beneath depict corresponding sagittal sections. **(D)** Quantification of *tbxt* expression rescued by *ets2* mRNA. **(E)** Circular *tbxt* expression of st. 11 specimen **(F)** faded upon unilateral dorsal *rab7* LOF (black arrowhead). **(G)** Coinjection of *mapk1* ^*D324N*^ mRNA in morphant embryos partially restored lost dorsal *tbxt* expression (black outlined arrowhead). **(H)** Quantification of *tbxt* expression rescued by *mapk1* ^*D324N*^ mRNA. **(I)** *myod1* horseshoe-shaped expression of st. 11 specimen **(J)** displayed downregulated paraxial transcripts after dorsal loss of *rab7* (black arrowheads). **(K)** *ets2* mRNA injected dorsally rescued the paraxial mesoderm identity (outlined black arrowheads); please note ectopic expression found in animal areas above the dorsal gap (white arrowhead). **(L)** Quantification of *myod1* expression rescued by *ets2* mRNA. **(M)** Horseshoe *myod1* expression of st. 11 specimen **(N)** faded upon dorsal *rab7* LOF (black arrowheads). **(O)** Coinjection of *mapk1* ^*D324N*^ mRNA in morphant embryos partially restored lost dorsal *myod1* expression (black outlined arrowheads). **(P)** Quantification of *myod1* expression rescued by *mapk1* ^*D324N*^ mRNA. **(Q)** Injection scheme and localization of imaged animal tissue of st. 12.5 embryos **(R,R′′)** Nucleic and cytosolic accumulation of pMapk1 (magenta) at posterior neural plate of wild type specimen. **(S,S′′)** Downregulation of pMapk1 signal intensity in *rab7*-morphant neural plates. **(R′,S′)** For lineage tracing and nuclear staining of *h2b* mRNA (green) was used, cell borders are highlighted by F-actin (blue). **(T)** Intensity quantification of pMapk1 signal in panels **(R,S)**. All *rab7* TBMO injections in this figure were done with high dose approach. a, anterior; co, control; d, dorsal; N, number of experiments; n, number of evaluated embryos; p, posterior; pmol, picomole; st., stage; TBMO, translation blocking Morpholino oligomere; v, ventral; wt, wild type; µm, micrometer.

Ets2 is the main transcription factor regulating mesoderm development downstream of FGF/MAPK signaling in *Xenopus* (Kawachi et al., 2003). It is sufficient to rescue loss of mesoderm specification caused by inhibition of the Fgf receptor (Kjolby et al., 2019). As reported before, dorsal injection of *ets2* mRNA was unable to activate *myod1* in the axial (notochordal) region of the early gastrula, but induced exogenous expression in anterior areas, demonstrating dose-efficiency (Supplementary Figures S1D–F; cf. Kjolby et al., 2019). However, it was sufficient to compensate the loss of *tbxt* in the axial mesoderm after dorsal loss of Rab7 (Figures 3A–D). The same effect was obtained when using an activated version of *Xenopus* MAPK (MAPK^D324N^; Umbhauer et al., 1995), which was able to partially rescue the dorsal loss of *tbxt* (Figures 3E–H). Importantly, both constructs were also able to rescue the loss of *myod1* in the paraxial mesoderm in mid-gastrula stages (Figures 3I–P). Confirming this hierarchical connection, Rab7 inhibition blocked endogenous Erk phosphorylation in the posterior animal cap/neural plate in late gastrula stages, as assessed using a well-characterized pErk1/2 antibody (Kinoshita et al., 2020; Figures 3Q–T). From these experiments we concluded that Rab7 function is necessary for correct MAPK-dependent specification of mesodermal and somitic identities during gastrulation.

### Rab7 is required for FGF-dependent mesoderm induction

**FIGURE 4:**
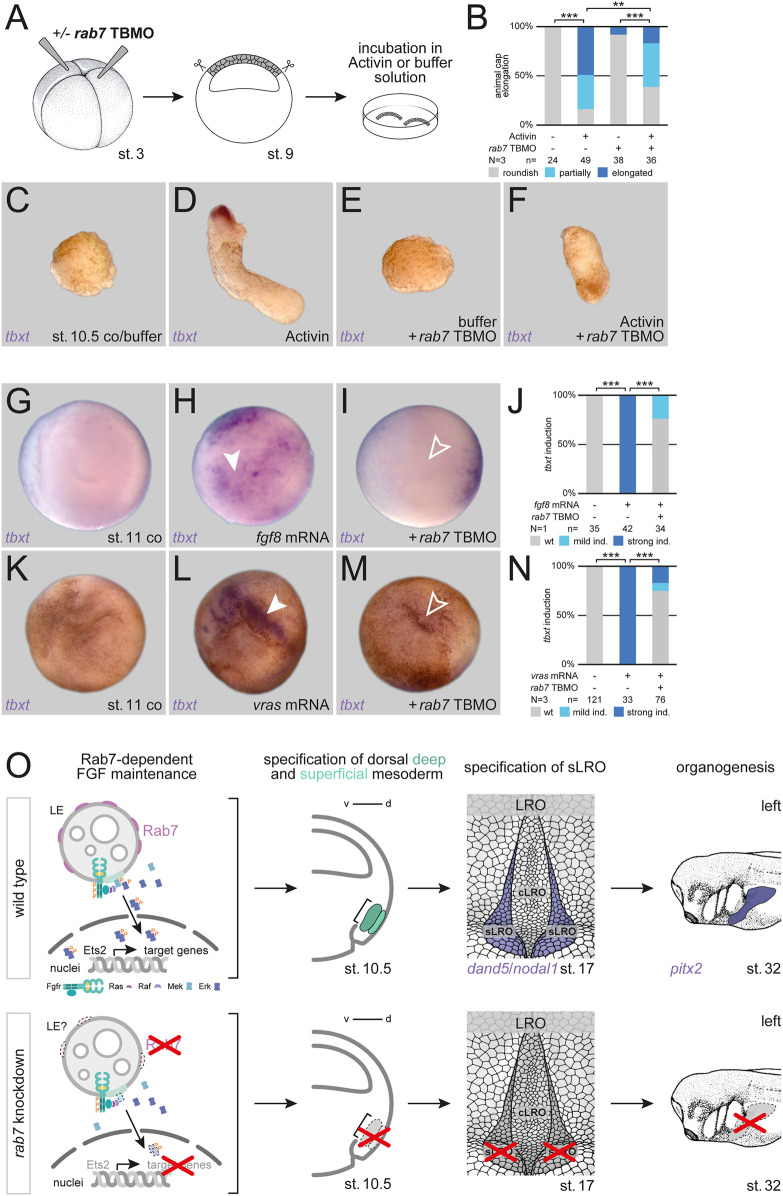
Rab7 is required for FGF-dependent mesoderm induction. **(A)** Experimental setup for the shown animal cap elongation assay: Injection at four-cell stage (left) targeted the animal hemisphere, at st. 9 animal caps were dissected (middle) and incubated in Activin A or buffer solution (right). **(B)** Quantification of animal cap elongation. **(C)** Untreated animal caps **(D)** elongated upon Activin treatment. **(E)** *rab7*-deficient, untreated animal caps remained in a roundish shape, **(F)** treated ones depicted partial or complete inhibition of elongation. **(D)** *tbxt* expression was only observed in Activin treated, elongated caps. **(G,K)** Wild type animal caps of st. 11 embryos. **(H)** Induction of *tbxt* expression (white arrowheads) in animal caps by *fgf8* or **(L)** *vras* mRNA overexpression. **(I,M)** Loss of *rab7* in those animal caps blocked *tbxt* induction (outlined arrowheads). **(J,N)** Quantification of *tbxt* induction *via fgf8* or *vras* mRNA. All *rab7* TBMO injections in this figure were done with high dose approach. **(O)** Proposed model for Rab7-dependent dorsal mesoderm and subsequent sLRO specification triggering LR development: (Top) Rab7-positive LEs could provide the platform for sustained FGF signal transduction (left). FGF signals mediate deep and superficial mesoderm specification (middle-left). Mesoderm development enables LRO morphogenesis (middle-right), the crucial tissue for LR asymmetry (right). (Bottom) Loss of Rab7 would inhibit FGF signal maintenance due to lost signaling platforms (left), causing a lack of deep and superficial mesoderm specification (middle-left). Loss of mesoderm prevents sLRO formation (middle-right) blocking LR axis establishment (right). cLRO, central LRO; co, control; d, dorsal; ind., induction; LRO, left-right organizer; N, number of experiments; n, number of evaluated embryos; sLRO, somitic LRO; st., stage; TBMO, translation blocking Morpholino oligomere; v, ventral; wt, wild type.

Induction of the frog dorsal mesoderm is mediated by TGFβ signals acting in concert with FGF receptor input. FGF signaling triggers MAPK activation, which is required for maintaining mesodermal identities in general, and for later axial elongation of dorsal mesodermal tissues (Dorey and Amaya, 2010). To test if Rab7 enables MAPK activation by participating in upstream intracellular FGF signal transduction, we first analyzed Activin A-mediated mesoderm induction in isolated animal caps by scoring elongation behavior, a process known to require active FGF signaling (Symes and smith, 1987; Cornell and Kimelman, 1994; Cornell et al., 1995). While Activin treatment induced elongation of control caps in most cases, this process was strongly inhibited in Rab7-deficient animal caps, i.e., recapitulating the lack of axial elongation we observed in Rab7-deficient embryos recently (Figures 4A–F; cf. Kreis et al., 2021). In support of this effect, Rab7 knockdown also blocked *nodal1*-mediated induction of an exogenous organizer in the animal hemisphere, and the circularly induced *tbxt* expression surrounding it, a whole-mount assay that requires FGF signaling as well (Supplementary Figures S2A–E; Kurth and Hausen, 2000). In this assay, it phenocopied the effect of a *dominant-negative fgf receptor 1* (Supplemenatry Figure S2F; Amaya et al., 1991). Next, we narrowed down the role of Rab7 for FGF signal transduction by inducing mesodermal fates in whole-mount animal hemispheres. Injecting mRNA coding the ligand Fgf8 or an activated mutant of the GTPase Ras (vRas) efficiently induced expression of *tbxt* in animal caps, indicating mesodermal fate. In both cases this induction was significantly inhibited in embryos additionally depleted of Rab7, suggesting a functional requirement of Rab7 downstream or at the level of Ras, i.e., for MAPK cascade initiation (Figures 4G–N). Thus, from these experiments we conclude that Rab7 participates in FGF-induced mesoderm specification during early gastrulation by regulating Ras-dependent MAPK activation.

## Discussion

### Rab7 deficiency recapitulates FGF-type LR phenotypes

LR axis formation follows, and is tightly linked to, correct establishment of the DV and AP axes, which means gastrulation and axial elongation are a prerequisite for subsequent establishment of a functional LRO, and thus, for LR symmetry breakage. Therefore, inhibiting these processes prevents analyses of LR features at later stages. As we had shown recently that loss of Rab7 causes such phenotypes during gastrulation (Kreis et al., 2021), we circumvented these early phenotypes by using specific targeting and reduced dose injections to be able to analyze LR axis defects. With this approach we here demonstrated that Rab7 is required for correct LR asymmetry by regulating LRO sensor morphogenesis. The observed phenotypes mimicked the loss of FGF signaling during mid/late gastrulation, which had also no early effect on general mesoderm specification but resulted in LR defects due to failed LRO sensor formation (Sempou et al., 2018; Schneider et al., 2019). With our approach, we found the paraxial mesoderm marker *myf5* to be more sensitive to a partial knockdown of Rab7 than the pan-mesoderm marker *tbxt*. In support of this observation, Sempou et al. described Fgf receptor 4 to be specifically required for paraxial mesoderm development and LR sensor formation during gastrulation, adding a further potential input for MAPK activation (Sempou et al., 2018). This correlates with the initial observation of Amaya and colleagues, who described exactly this paraxial sensitivity when using a *dn-fgfr1* construct (Amaya et al., 1993). Importantly, very recently Myf5 has been demonstrated to be required for LRO sensor formation in *Xenopus*, directly linking somitogenesis to LR asymmetry (Tingler et al., 2022). Thus, inhibition of *myf5* is sufficient to explain the LR defects obtained by loss of Rab7. In that report *myf5* was shown to be regulated by the somitogenesis transcription factor Dmrt2 (Doublesex and Mab-3 Related Transcription Factor 2). In future, it will be interesting to see if Rab7/FGF acts upstream of Dmrt2, or if both represent parallel regulatory inputs on *myf5*.

The same FGF similarities were true for specification of the LRO precursor, indicated by *foxj1* expression in the SM layer during early gastrulation. In parallel to blocking mesoderm induction, early inhibition of FGF inhibited *foxj1* expression without touching organizer specification, finally leading to massive gastrulation defects and lack of axial elongation (Amaya et al., 1993; Fletcher and Harland, 2008; Schneider et al., 2019). We found the same phenotypes in our recent study after knockdown of Rab7, which also resulted in strong inhibition of *foxj1*, in parallel to reduction of mesodermal and myogenic markers like *tbxt*, *myf5*, *tbx6*, and *myod1*, i.e. phenocopying an early loss of FGF (Kreis et al., 2021). In addition to FGF, induction of *foxj1* in the SM is also dependent on early canonical Wnt signaling (Beyer et al., 2012; Walentek et al., 2013). In our previous work, we were able to connect the loss of Rab7 in the ventro-lateral mesoderm to impaired Wnt signaling. However, we found no evidence that loss of dorsal SM specification (i.e., *foxj1* expression) is linked to a loss of early dorsal Wnt signals at blastula/early gastrula stages (Kreis et al., 2021). In *rab7*-deficient conditions, endogenous organizer genes were still expressed comparable to wild type. Moreover, expression of the canonical Wnt target gene *nodal3*, which is expressed in the SM and codes for an atypical TGFβ ligand, was barely touched upon loss of Rab7 (Smith et al., 1995; Glinka et al., 1996; Kreis et al., 2021). Importantly, Nodal3 is thought to activate Fgfr1 and has been shown to be required upstream of (dorsal) *tbxt* and *foxj1* expression, i.e., regulating mesoderm maintenance and SM specification in parallel. Finally, Nodal3 knockdown caused a comparable phenotype to inhibition of Rab7 or Fgfr1 (Amaya et al., 1991; Yokota et al., 2003; Vick et al., 2018; Kreis et al., 2021). Therefore, Rab7 is most likely required for *foxj1* expression in a FGF-dependent manner, but independent of Wnt signaling.

### FGF-dependent mesodermal patterning depends on Rab7

In our second set of experiments we presented evidence that Rab7 modulates intracellular transduction of FGF signals during gastrulation, most likely at the level of activating Ras or downstream Raf kinases. As mentioned above, the knockout or knockdown phenotype of Rab7 phenocopies that of a dominant-negative Fgf receptor 1 (Amaya et al., 1991, 1993; Kreis et al., 2021), or that of Nodal3 (Yokota et al., 2003), both of which are required for correct *tbxt* expression and gastrulation/axial elongation in *Xenopus*. Expression of *rab7* in these stages is restricted to the deep mesoderm, very reminiscent of *tbxt* (Kreis et al., 2021). As said, *nodal3* and *foxj1* are restricted to the superficial layer, while *fgfr1* is found in both areas, deep and superficially (Smith et al., 1995; Stubbs et al., 2008; Yamagishi and Okamoto, 2010). Finally, Fgf receptor 4 signaling has been shown to be required specifically for paraxial mesoderm formation during gastrulation (Sempou et al., 2018). Together this suggests a diverse set of autocrine and paracrine routes taken by potential ligands. Superficial Nodal3 secretion, or a putative Fgf ligand (e.g., Fgf4) secreted from deep cells could both act on both tissues, i.e., impacting *foxj1* superficially and *tbxt* in the deep. However, as from our data Rab7 acts in intracellular MAPK activation, i.e., in the responding cell, it should regulate *tbxt* directly but *foxj1* in an indirect manner, potentially by interfering with the known Fgf4-Tbxt feedback loop (Schulte-Merker and Smith, 1995).

### Late endosomal regulation of MAPK activity and the potential role of Rab7

From our analyses we conclude that Rab7 participates in signal transduction generated by FGF receptor activation at the level or downstream of Ras. But how could Rab7 influence this cellular transduction event mechanistically? As a very important regulator of LE and lysosomes, Rab7 exerts its cellular roles mostly by controlling maturation and trafficking of LE (Guerra and Bucci, 2016). In theory it would enable degradation of any activated receptor complex and thus regulate signaling output in a negative manner (Platta and Stenmark, 2011). However vice versa, to our knowledge no positive involvement of LE in FGF-induced signaling has been presented so far. Yet, in context of Rab7-mediated membrane trafficking, it is important to notice that robust Erk/MAPK activation has been reported to be associated with LE adaptors. While EGF-induced early activation of Erk at the plasma membrane did not dependent on Lamtor adaptor complex, sustained activation of Erk was only observed when this complex was bound to its target membranes, namely mature LE (Teis et al., 2002, 2006; Nada et al., 2009). Furthermore, EGF receptor endocytosis, its endosomal trafficking, and concomitant recruitment of Ras and Raf kinase to Rab7-positive late endosomal structures fostered colocalization with the Lamtor complex (Lu et al., 2009). MEK1, in turn, specifically binds to its scaffold MEK 1 partner (MP1/Lamtor3), bridging it also to LEs, which finally leads to its phosphorylation by Raf (Teis et al., 2002; Nada et al., 2009; Tebar et al., 2018).

Therefore, in the context of FGF-dependent mesoderm patterning, inhibition of Rab7 should theoretically disrupt LE maturation, which could prevent LE-dependent activation of MAPK. We therefore propose that mesodermal FGF signaling in *Xenopus* also relies on this late endosomal mechanism to maintain MAPK in an active state during mesoderm development (Figure 4O). A higher level (or longer maintaining) requirement of MAPK signal could also explain the higher sensitivity of the paraxial mesoderm for disrupting FGF receptor signaling or Rab7 inhibition (Amaya et al., 1993 and this work). Finally, if canonical Wnt (Taelman et al., 2010; Vinyoles et al., 2014) and FGF/MAPK (this work) signaling indeed both relied on LEs to maintain robust signal outputs, this could be a different mechanism of positively integrating both pathways, which are known to interact in several contexts (see for example, McGrew et al., 1997; Dorey and Amaya, 2010; Kjolby et al., 2019).

## Materials and methods

### *Xenopus laevis* care and maintenance

Frogs were purchased from Nasco (901 Janesville Avenue P.O. Box 901 Fort Atkinson). All handling, care, and experimental manipulations of animals was approved by the Regional Government Stuttgart, Germany (V349/18ZO ‘Xenopus Embryonen in der Forschung’), according to German regulations and laws (§6, article 1, sentence 2, nr. 4 of the animal protection act).

### Microinjection setup

For microinjections, a Harvard Apparatus setup was used and drop size was set to 4 nl per injection. For lineage specific verification fluorescein dextran, *mGFP* mRNA, *h2b* mRNA or *lacZ* mRNA was coinjected and validated as described earlier (Kreis et al., 2021). Embryos were injected at the four-cell stage either in the animal hemisphere or in the dorsal marginal zone.

### Microinjections

The rab7a TBMO was used as published (Kreis et al., 2021); sequence is 5′-GTC​TCC​GCT​TCC​TAC​CCC​TGC​CAG​C-3′. A random coMO was used as a MO fill up in this study. Total amounts of injected *rab7* TBMO was: 0.4 pmol (for high dose approach) or 0.8 pmol (for low dose approach) per blastomere, as indicated in the figure legends. For mRNA synthesis plasmids were linearized with NotI and transcribed *in vitro* (Sp6 polymerase) using Ambion message machine kit. Total amounts of injected mRNA per embryo are as follows: 80 pg *dn-fgfr1* mRNA, 400 pg *ets2* mRNA, 400 pg *fgf8* mRNA, 400 pg *h2b-GFP* mRNA, 400 pg *lacZ* mRNA, 12–20 pg *mapk1* ^*D324N*^ mRNA, 400 pg *mGFP* mRNA, 20 pg *nodal1* mRNA, 8 pg *vras* mRNA.

### Immunofluorescence analysis

For IF analyses, *h2b-GFP* mRNA was coinjected as lineage tracer. After reaching the appropriate stage, embryos were fixed in MEMFA for 1 h at room temperature, this was followed by 2 washes in 1x PBS- for 15 min. Afterwards whole mounts were transferred to 24 well plates and washed two times for 15 min in PBST (PBS/0.1% Triton X-10). Then blocking for 2 h at room temperature in CAS-Block (1 : 10 in PBST; ThermoFisher, # 008120) was performed, blocking reagent was replaced by antibody solution (diluted in CAS-Block) for incubation over night at 4 °C. Primary antibody used was anti-pMapk1/Erk (Cell Signaling, # 4370, 1: 200). Antibody solution was removed by washing twice for 15 min in PBS. Secondary antibody (ThermoFisher, all 1: 1000 in CAS-Block) was applied for 2 h at room temperature or overnight at 4°C. F-actin was directly visualized using AlexaFluorTMPlus 405 Phalloidin (ThermoFisher, A30104; 1: 200 in PBS-) for cell boarder detection. Photo documentation was performed with whole mounts or animal caps being transferred to microscope slides.

### Animal cap elongation assay

rab7 TBMO was injected 2x at the four-cell stage into the animal hemisphere. Embryos were raised until stage 9 in 0,1x MBSH solution. Animal caps were manually dissected and individually transferred to 24 well plates, containing an Activin A solution (10 ng/mL in 0,5x MBSH) or buffer and incubated until stage 13–15. Used well plates have been coated with 2% BSA (bovine serum albumin) in 1x PBS- (phosphate buffered saline; pH = 7,4). For subsequent *in situ* hybridization and photo documentation animal caps were fixed 2–3 h at room temperature in MEMFA.

### *In situ* hybridization

RNA *in situ* probes were synthesized by using SP6 or T7 polymerases. Embryos were fixed 2–3 h at room temperature for *in situ* mRNA detection and further processed according to a customized (after R. Rupp, personal communication) standard protocol.

### Embryo sections

Bisections of embryos were performed by using a razor blade. For more high-quality sections embryos were embedded in a glutaraldehyde-crosslinked gelatin-albumin mix and sectioned with a vibrating blade microtome.

### Photo documentation

LSM images of IF data were taken with a Zeiss LSM 700 Axioplan2 Imaging microscope and then adjusted using the Zeiss Zen 2012 Blue edition. All other pictures were taken with a Zeiss SteREO Discovery. V12 microscope or an Axioplan2 inverted microscope using AxioVision 4.6. Afterwards Adobe Photoshop was used for cropping and careful brightness adjustments.

### Statistical analysis

Any probability of the collected data was calculated with Pearson’s chi-square test (Bonferroni corrected, if required). Significance levels of all statistical analyses were grouped as follows: * = *p* < 0.05, ** = *p* < 0.01, *** = *p* < 0.001. *N* = or *n* = declares number of experiments or number of embryos analyzed, respectively.

## Data Availability

The original contributions presented in the study are included in the article/Supplementary Material, further inquiries can be directed to the corresponding author.
