# Supplementary material for: FGF-mediated establishment of left-right asymmetry requires Rab7 function in the dorsal mesoderm in Xenopus
Source: Front Cell Dev Biol. 2022 Aug 29;10:981762. doi: 10.3389/fcell.2022.981762 (PMC9465294; doi:10.3389/fcell.2022.981762)
Supplement: Supplementary file 1 [file DataSheet1.PDF]

Supplementary Material

Supplementary Figures

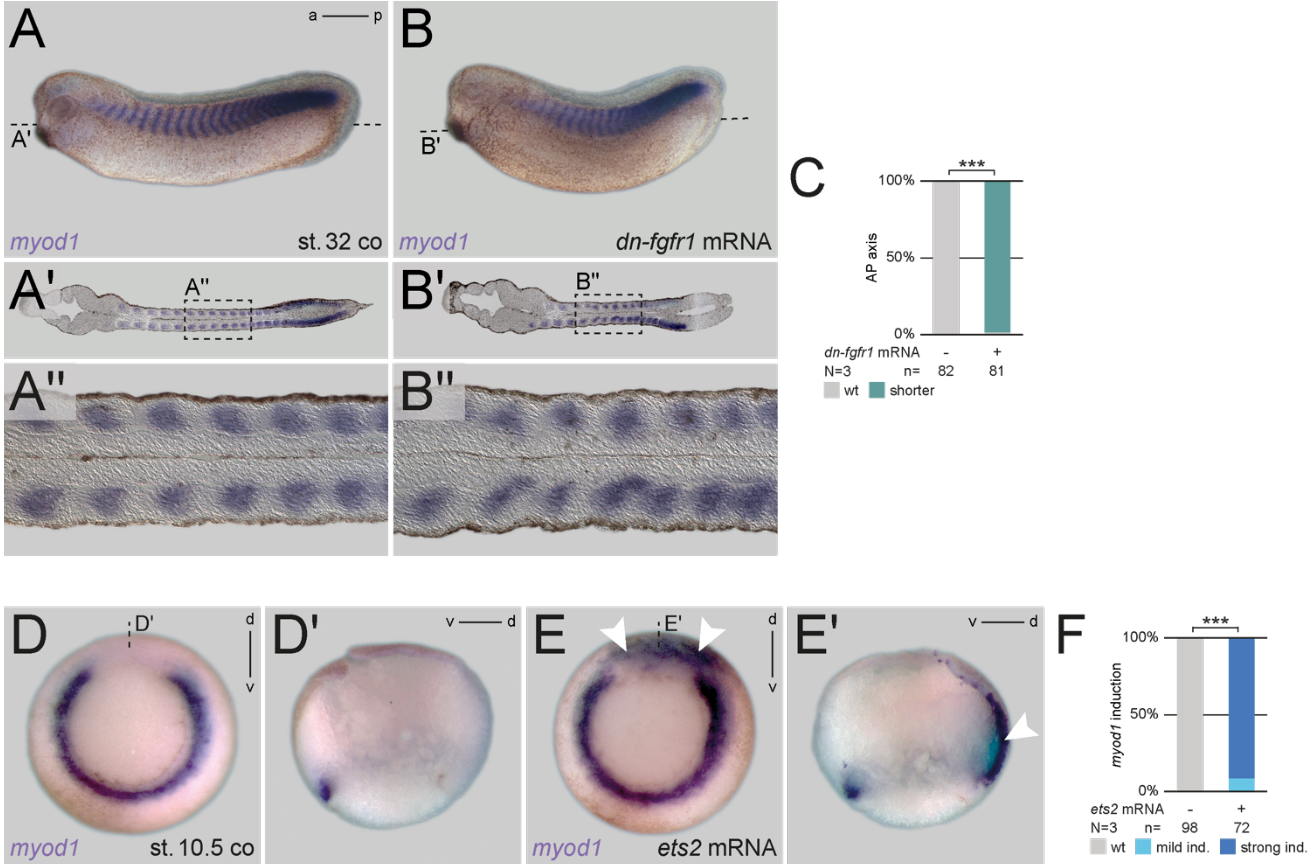

Supplement Figure 1. Loss of Rab7 blocks dorsal mesoderm specification upstream of Mapk activation.

(A) *Xenopus* st. 32 tadpoles depicted wildtype axis elongation compared to (B) shortened axis of *dn-fgfr1* mRNA treated specimen, somites are highlighted by *myod1* expression. (A', B') Frontal sections through somitic domain, (A'', B'') zoom-ins of *myod1* expressing somites as indicated in dashed boxes, respectively. (C) Quantification of axis phenotype. (D) Dorsal gap of wildtype *myod1* expression of st. 10.5 embryos (E) depicted ectopic *myod1* induction upon *ets2* mRNA overexpression (white arrowheads), (D', E') clarified in sagittal sections respectively. (F) Quantification of dorsal *myod1* induction.

a, anterior; co, control; d, dorsal; ind., induction; N, number of experiments; n, number of evaluated embryos; p, posterior; st., stage; TBMO, translation blocking Morpholino oligomere; v, ventral; wt, wildtype.

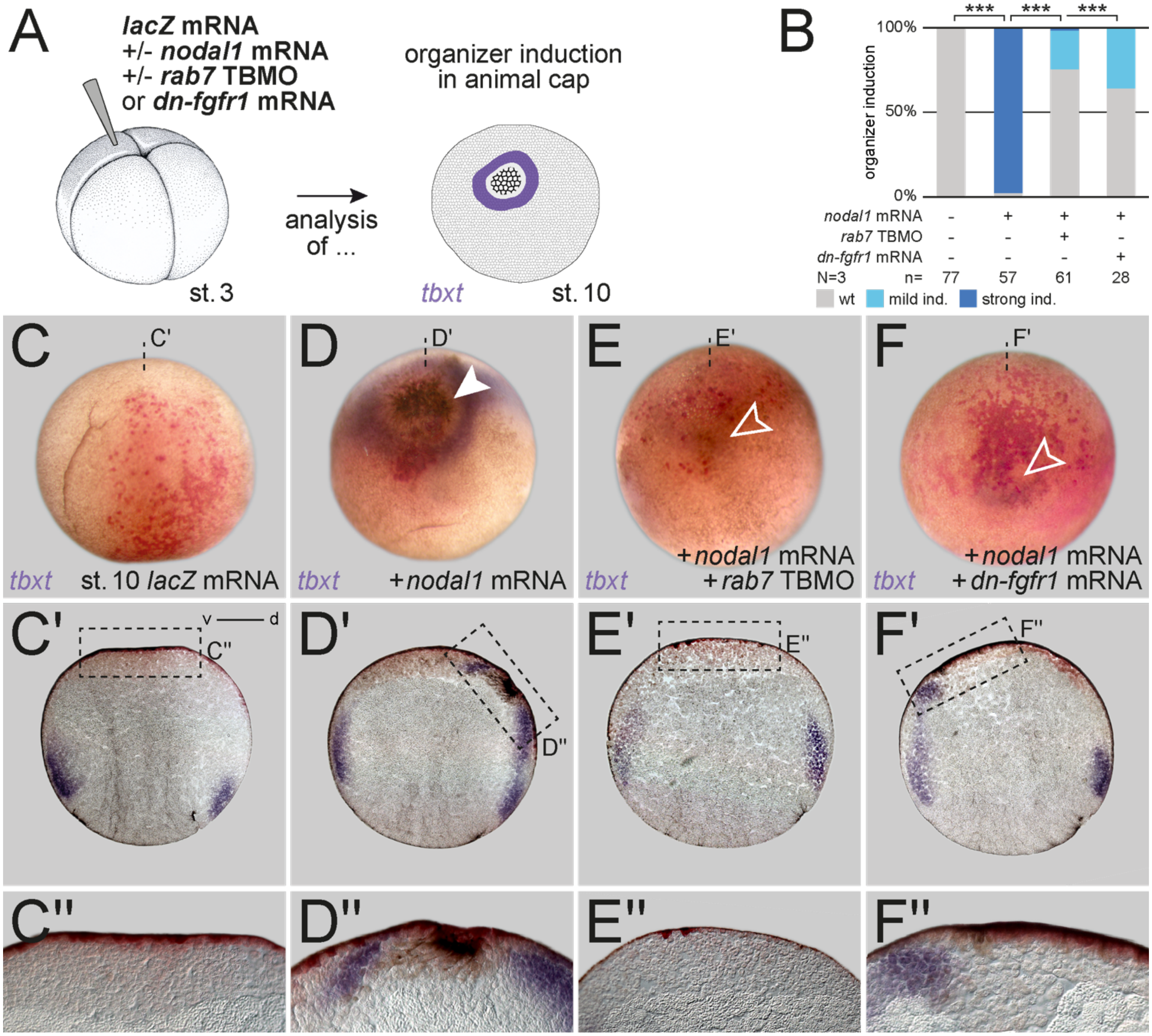

Supplement Figure 2. Dorsal mesoderm induction depends on Rab7.

(A) Experimental setup of artificial organizer induction: Injection at four-cell stage (left) targeted animal hemisphere to analyze artificial organizer induction and surrounding *tbxt* expression in animal caps of st. 10 specimens. (B) Quantification of induced artificial organizers in animal caps. (C) Wildtype animal caps of st. 10 embryos. (D) *nodal1* mRNA injection induced pigment accumulation, indicating organizer induction (white arrowhead), induction site is sphered by *tbxt* expression. (E, F) Co-injection of either *rab7* TBMO or *dn-fgfr1* mRNA resulted in artificial organizer inhibition (outlined white arrowheads) and blocked *tbxt* expression in animal caps. (C'-F') Panels beneath show half sections of treated tissues and (C''-F'') zoom-ins of potential organizer induction sites, respectively. (C-F) For lineage tracing *lacZ* staining was used.

co, control; d, dorsal; ind., induction; N, number of experiments; n, number of evaluated embryos; st., stage; TBMO, translation blocking Morpholino oligomere; v, ventral; wt, wildtype.
